# Changes in macrophage and inflammatory cytokine expressions during fracture healing in an ovariectomized mice model

**DOI:** 10.1186/s12891-021-04360-z

**Published:** 2021-05-28

**Authors:** Lin Chen, Shao Cheng, Kanghui Sun, Jing Wang, Xinhua Liu, Yongjian Zhao, Junjie Yang, Dongfeng Zhao, Chunchun Xue, Yuren Tao, Shitian Zhao, Hao Zhang, Sheng Lu, Qi Shi, Yongjun Wang, Bing Shu

**Affiliations:** 1 Longhua Hospital, Shanghai University of Traditional Chinese Medicine, Shanghai, 200032 China; 2 Spine Institute, Shanghai Academy of Traditional Chinese Medicine, Shanghai, 200032 China; 3 Key Laboratory, Ministry of Education of China, Shanghai, 200032 China; 4 Shanghai Municipal Hospital of Traditional Chinese Medicine, Shanghai University of Traditional Chinese Medicine, Shanghai, China; 5 Experiment Center for Science and Technology, Shanghai University of Traditional Chinese Medicine, Shanghai, 201203 China

**Keywords:** Ovariectomy, Fracture healing, Macrophage, Tumor necrosis factor-α, Interleukin-6

## Abstract

**Background:**

Macrophages and inflammatory cytokines play important roles in bone fracture healing. However, the expression patterns of macrophages and inflammatory cytokines during fracture healing under the condition of postmenopausal osteoporosis have not been fully revealed.

**Methods:**

Tibia transverse fracture was established 12 weeks after ovariectomy or sham operation in 16-week old female mice. Tibias were harvested before fracture or 1, 3, 5, 7, 14, 21, 28 days after fracture for radiological and histological examinations. M1/M2 inflammatory macrophages, osteal macrophages and gene expressions of tumor necrosis factor-α, interleukin-6, interleukin-1β and macrophage conversion related molecules in the fracture haematoma or callus were also detected.

**Results:**

The processes of fracture healing, especially the phases of endochondral ossification and callus remodeling, were delayed in ovariectomized mice. The expressions of tumor necrosis factor-α and interleukin-6, but not interleukin-1β, in the fracture haematoma or callus were disturbed. Expressions of tumor necrosis factor-α were decreased at 1, 14 and 21 days post-fracture (DPF), and were increased at 3, 5 and 7 DPF. Interleukin-6 expressions at 1, 3 and 21 DPF were significantly increased. We found the decreases in M1 and M2 macrophages at 1 DPF of the initial inflammatory stage. M2 macrophages at 14 DPF of the middle stage and osteal macrophages at 14, 21 and 28 DPF of the middle and late stages of fracture healing were also reduced in ovariectomized mice.

**Conclusions:**

The expressions of macrophages and inflammatory cytokines were impaired in ovariectomized mice, which might contribute partially to poor fracture healing.

**Supplementary Information:**

The online version contains supplementary material available at 10.1186/s12891-021-04360-z.

## Background

The process of fracture healing consists of inflammation stage, soft callus stage, hard callus stage and remodeling stage. Inflammation reaction in the initial phase of fracture which contains macrophages and inflammatory cytokines has been recognized critical for fracture healing. These inflammatory cells secrete a large number of cytokines which has been proved to play an important role in cell recruitment and angiogenesis as well as bone repair [1].

Macrophages can be categorized into inflammatory macrophage and resident macrophage. Inflammatory macrophages can be further classified into classically activated M1 phenotype and alternatively activated M2 phenotype, which can be shifted into each other under certain conditions [2]. In general, M1 macrophages produce inflammatory cytokines such as tumor necrosis factor-α (TNF-α), interleukin-1β (IL-1β) and interleukin-6 (IL-6), and mediate inflammatory response, while M2 macrophages secret interleukin-10 and interleukin-1 receptor antagonist, and mediate tissue repair and resolution of inflammation [3]. Inflammatory macrophages are central mediators of the inflammatory response, the dynamic changes and balance between macrophage M1/M2 play important roles in inflammatory regulation and tissue repair. Different from inflammatory macrophages, resident macrophages are present in all tissue with tissue-specific phenotypes and functional abilities [4, 5]. Resident macrophages in bone tissue located on periosteal and endosteal surface are termed osteal macrophages [6]. Studies have demonstrated that inflammatory macrophages were highly presented in the early anabolic phase; while osteal macrophages mature the bone callus, predominating in the late anabolic and remodeling phases during fracture healing [7, 8].

Macrophages and inflammation cytokines are proved to play important roles in callus formation, cartilage deposition and callus remodeling during bone fracture healing [7, 9–12]. Several studies have proved that the dysregulation of inflammatory environment during bone fracture could impair fracture healing, which was seen in diabetics, obesities and smokers [13–15]. Menopause, one of the most important factors in the pathogenesis of postmenopausal osteoporosis, was considered to induce a systemic chronic inflammatory reaction with the increase of TNF-α, IL-1β, IL-6 which were the major inflammatory factors during fracture healing [16]. Fracture healing in animal models of postmenopausal osteoporosis revealed a decrease in bone callus formation in the early stage, delay of endochondral bone formation and hard callus remodeling in the middle and late stages [17, 18]. In addition, the pathological histology of postmenopausal osteoporotic fracture was similar to fracture with abnormal expression of macrophages or inflammatory cytokines [17, 18].

A systematic review analyzed the differences in inflammatory responses in normal and osteoporotic fractures, and found that it was inconclusive whether OVX animals have higher or lower local inflammatory response, and declared that there was a need for further studies to better understand the role of the inflammatory response in the healing cascade for potential immunomodulation to enhance osteoporotic fracture healing [19]. A recent study found that OVX impaired the innate immune response locally at the fracture site in rats. However, there was no study observing the dynamic changes in inflammatory response during osteoporotic fracture healing systematically, especially in the inflammation stage [20]. To address this issue, in this study, we established tibia fracture model in ovariectomized (OVX) mice, and observed the changes in macrophages and inflammatory cytokine expressions during different stages of fracture healing.

## Methods

### Animal experimentation

All applicable institutional and national guidelines for the care and use of animals were followed. The experimental protocols were performed with the approval of Institutional Animal Care and Use Committee of Shanghai University of Traditional Chinese Medicine (PZSHUTCM18121414). Female C57BL/6 mice were purchased from Charles River Company (Beijing, China). The mice were housed in environmentally controlled animal facilities at 22 °C and humidity of 50 to 60%, with a 12 h light/dark cycle, and were fed a commercial diet and distilled water ad libitum. The maximum caging density was five mice.

Following a 1-week acclimatization phase, 210 16-week-old mice were randomly assigned to be bilaterally ovariectomized or to undergo sham surgery. Random numbers were generated using the standard = RAND() function in Microsoft Excel. Four mice in the OVX group and 3 in the sham group died after ovariectomy or sham surgery. Twelve weeks after the surgery, mice were performed a mid-shaft transverse osteotomy fracture or sham surgery on the left tibias, and the tibias were fixed with 0.5-mm-diameter intramedullary metallic pins. Another 14 mice, including 6 mice in the sham group and 8 in the OVX group, were excluded from following observations because comminuted fractures were made. Another 3 mice were used to supplement the sample size of the OVX group. All the mice were anaesthetized by intraperitoneal injection of ketamine (67 mg/kg) and xylazine (5 mg/kg) before fracture. After fracture, mice were allowed spontaneous recovery in warmed cages and unrestricted weight bearing. Mice were given Buprenorphine (0.1 mg/kg) for 3 days to relief any pain.

Mice in each group were sacrificed by exsanguination and confirmation of death by cervical dislocation before fracture or 1, 3, 5, 7, 14, 21, 28 days post-fracture (DPF) (*n* = 12 at each time point). Serum was separated for estradiol (E2) detection. The left tibias (6 from each group per time point) were harvested and fixed in 10% buffered formalin for 24 h. After fixation, the specimens were washed by phosphate buffered saline, and stored in 75% ethanol at 4 °C for X-ray and micro-CT scanning. Following that, specimens were decalcified with 14% ethylene diaminetetraacetic acid disodium salt solution for 14 days, dehydrated and embedded. Four-μm thick serial sections were cut for histomorphometric study. The rest 6 tibias of each group were harvested for real-time polymerase chain reaction analysis (PCR).

### Blinding

For each mouse, different investigators were involved as follows: 2 investigators (JW and XL) administered the random allocation of the mice, and 5 investigators (DZ, CX, YT, SZ and HZ) performed bilaterally ovariectomy. Another 5 investigators (LC, SC, KS, YZ and JY) performed fracture and sacrifice procedures. Each investigator was responsible for part of the surgeries, including anesthesia, skin preparation and disinfection, model establishment, suturing, and resuscitation of the mice. LC, SC and KS assessed the results of the experiment. Two investigators (JW and SL) were responsible for statistical analysis of the data.

### Serum E2 detection

Blood of mice in 0 DPF group was collected, and serum was separated after centrifugation at 3000 rpm for 15 min at room temperature. Serum concentrations of E2 were measured using a commercially available kit (ml058533, MLBIO, China) according to the manufacturer’s instructions.

### Three-dimensional (3D) reconstruction analyses

The fractured tibias underwent X-ray photography first. A five-point radiographic scoring system was used to quantify fracture healing status at each time point by two researchers separately, and the average scores were adopted. The intramedullary metallic pins in the fractured tibias were then removed, and underwent scanning using a micro-CT imaging system (μCT80, Bassersdorf, Switzerland) with 10 mm slice increment. The source voltage was 55 kV and the source current was 72 μA. The integration time was 300 ms. A reconstruction of the bitmap data set was used to construct the 3D images of the fracture site using the built-in software (Scanco Holding AG, Brüttisellen, Switzerland). Total volume (TV, mm^3^), bone volume (BV, mm^3^) and the ratio of bone volume to total volume (BV/TV, %) of the callus were also analyzed.

### Histological examination

Alcian blue hematoxylin/orange G (ABH/OG) staining was used to evaluate the histological changes of callus at different time points. Briefly, dewaxed sections of each group were stained with alcian blue/hematoxylin solution for 30 min followed by a 15-s wash in 1% ammonia solution and another 1-min wash in 95% ethanol solution. The sections were then incubated in orange G/eosin solution with phloxine B for 1 min. After dehydration in ascending series of ethanol solution, clearing in xylene and mounting, all sections were analyzed by an Olympus VS120-S6-W slide loader system (Olympus, Japan).

### Antibodies

The antibodies used for immunohistochemistry staining and immunofluorescence staining were purchased from Abcam (Shanghai, China) including anti-TNF-α antibody (ab6671, 1:200), anti-IL-1β antibody (ab9722, 1:400), anti-IL-6 antibody (ab83339, 1:2000), anti-F4/80 antibody (ab6640, for immunohistochemistry staining, 1:200), anti-F4/80 antibody (Alexa Fluor 488, ab204266, for immunofluorescence staining, 1:400), anti-inducible nitric oxide synthase (iNOS) antibody (Alexa Fluor 647, ab209027, 1:200), anti-Mannose receptor antibody (anti-CD206, Alexa Fluor 647, ab195192, 1:200), anti-Galectin 3 (Mac-2) antibody (ab53082, 1:200).

### Immunohistochemistry staining

Immunohistochemistry staining was used to evaluate the expressions of TNF-α, IL-1β, IL-6, F4/80 and Mac-2 in the fracture haematoma or callus. Deparaffinized and rehydrated serial sections were successively treated with 0.1% trypsin solution and 3% H_2_O_2_ solution for 15 min at 37 °C. Sequentially the sections were incubated in diluted primary antibodies overnight at 4 °C. Negative control sections were incubated in corresponding IgG solution instead. Then sections were incubated with secondary antibody and horseradish peroxidase (HRP)-streptavidin as instructed by the manufacturer (Polink-2 plus polymer HRP detection system kit, PV-9001/ PV-9004, ZSGB-BIO, Beijing, China). After staining with diaminobenzidine and counterstaining with hematoxylin, the images of the sections were obtained by an Olympus VS120-S6-W slide loader system, and were analyzed with an image Pro Plus 6.0 software (Media Cybernetics, PA, USA). Quantification of osteal macrophages (F4/80^+^ Mac-2^−^) was performed referring to the previous study [7]. For each section of the haematoma or callus, 6 regions were randomly selected for quantitative analysis, and the average value was calculated. For each group, 6 samples were included for statistical analyses.

### Immunofluorescence staining

To identify different types of macrophages in the fracture haematoma or callus tissue, dual immunofluorescence staining was performed with deparaffinized and rehydrated sections. Immunofluorescence staining was performed as described previously [21]. Briefly, sections were incubated with anti-F4/80 and anti-iNOS antibodies to identify M1 phenotypic macrophages; or incubated with anti-F4/80 and anti-CD206 antibodies to identify M2 phenotypic macrophages [22]. Finally, the sections were mounted in mounting medium with 4′,6-diamidino-2-phenylindole (DAPI, Ca H-1200, Vector, USA) and the images were obtained and analyzed by an Olympus VS120-S6-W slide loader system. For quantitative analysis of each section, positive stained cells of six random fields were counted and the average number of positive stained cells was calculated.

### Real-time PCR analysis

Total RNA was isolated from the tissue located 2 mm distal and proximal to the fracture site. Reverse transcription was performed using a Primescript™ RT reagent Kit (Takara Bio, Shanghai, China) and real-time PCR was performed in a total volume of 20 μl solution containing 10 μl SYBR Premix EX Taq (Takara Bio, Shanghai, China), 1 μl diluted cDNA, 10pM forward and reverse primers. Primers specific for the genes were listed in Table 1. The mRNA expression of each gene was normalized to β-actin. The experiments were repeated at least three times.

**Table 1:** Sequences of PCR primers for specific genes

Gene		Sequences
*β-actin*	Forward	5′-GGAGATTACTGCCCTGGCTCCTA-3’
	Reverse	5′-GACTCATCGTACTC CTGCTTGCTG-3’
*Tnf-α*	Forward	5′-CCTGTAGCCCACGTCGTAG-3’
	Reverse	5′-GGGAGTAGACAAGGTACAACCC-3’
*Il-1β*	Forward	5′-GGAGATTACTGCCCTGGCTCCTA-3’
	Reverse	5′-GACTCATCGTACTCCTGCTTGCTG-3’
*Il-6*	Forward	5′-GACAAAGCCAGAGTCCTTCAGA-3’
	Reverse	5′-GTCTTGGTCCTTAGCCACTCC-3’
*Cd16*	Forward	5′-CAGAATGCACACTCTGGAAGC-3’
	Reverse	5′-GGGTCCCTTCGCACATCAG-3’
*Cd206*	Forward	5′-GGAAACGGGAGAACCATCAC-3’
	Reverse	5′-GGCGAGCATCAAGAGTAAAG-3’
*Mcsf*	Forward	5′-AACAGCTTTGCTAAGTGCTCTA-3’
	Reverse	5′-ACTTCCACTTGTAGAACAGGAG-3’
*Mcp-1*	Forward	5′-TTTTTGTCACCAAGCTCAAGAG-3’
	Reverse	5′-TTCTGATCTCATTTGGTTCCGA-3’
*Il-4*	Forward	5′-AGT GAG CTC GTC TGT AGGGC-3’
	Reverse	5′-CAGGCA TCG AAA AGC CCG AA-3’

### Statistical evaluation

All data were presented as mean ± standard deviation and were analyzed by GraphPad Prism statistical software (version 6.01, California Corporation, America) as appropriate. Data sets was performed a Shapiro-Wilk test for normality and confirmed a comparison of variances test for Homogeneity. ANOVA with Turkey’s post hoc test or unpaired Student’s t tests was performed for comparison between two groups. *P* value < 0.05 was considered statistically significant.

## Results

### Fracture healing was delayed in OVX mice

**Fig. 1:**
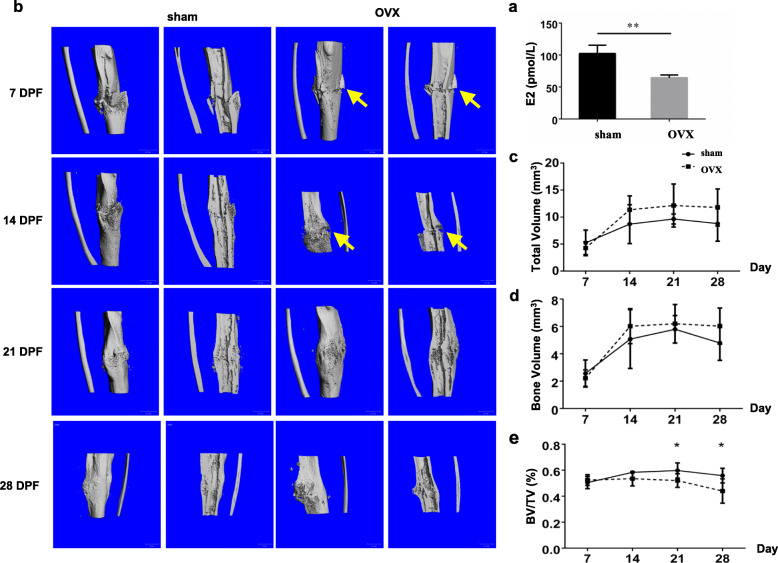
Delayed fracture healing and impaired callus structure in OVX mice. **a** Serum concentrations of E_2_ of OVX mice and sham mice at 0 DPF. **, *p* < 0.01 compared with the sham group. *n* = 10/group. **b** Three-dimensional reconstructions of the callus of sham and OVX mice at 7, 14, 21, and 28 DPF. DPF, days post-fracture. Arrows, fracture gap. **c**-**e** TV, BV and BV/TV of the callus at 7, 14, 21 and 28 DPF were quantified. *, *p* < 0.05 compared with the sham group. *n* = 6/group. OVX, ovariectomized; E_2_, estradiol; DPF, days post-fracture; TV, total volume; BV, bone volume

To confirm the establishment of OVX model, serum concentrations of E2 of OVX mice and sham mice were detected at 0 DPF, which was 12 weeks after ovariectomy. E2 concentration of OVX mice was obviously decreased compared with that of sham mice (Fig. 1a).

To reveal the morphological differences in callus between OVX mice and sham mice, 3D reconstructions (Fig. 1b) were performed. There was no obvious difference between the two groups until 14 DPF. At this time point, callus formation of the sham mice was evident with bridge connecting the fracture gap and the fracture line was almost invisible. However, the fracture gap was still obvious in OVX mice. In addition, the callus of sham mice became small at 21 and 28 DPF, while it was still obvious in OVX mice. These results were consistent with the X-ray findings (Additional Figure 1) and suggested a delayed fracture healing process in OVX mice. Quantitative analyses showed that both callus TV and BV of OVX group were increased in the tendency compared with that of sham group, though no significance was shown (Fig. 1c and d). BV/TV values (21 and 28 DPF) of OVX group were significantly decreased (Fig. 1e, *p* < 0.05), indicating the bone mass of healed tibias in OVX mice was decreased.

### Endochondral ossification and callus remodeling were impaired in OVX mice

**Fig. 2:**
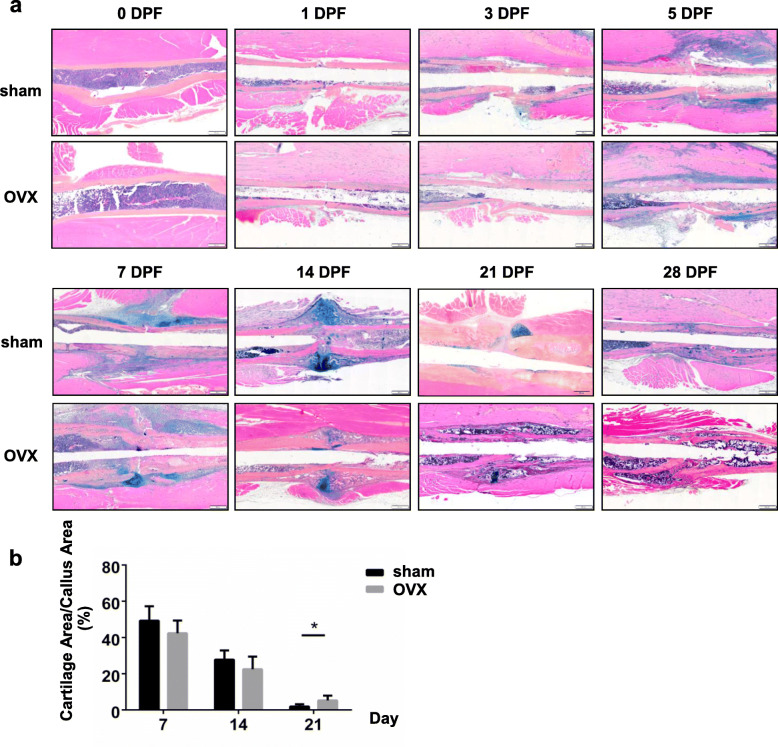
Impaired endochondral ossification and callus remodeling in OVX mice. **a** ABH/OG staining of the callus in sham and OVX mice at different time points of fracture healing. Scale bars, 500 μm. **b** Quantification of the percentages of cartilage area in total callus area. *, *p* < 0.05 compared with the sham group. *n* = 6/group. OVX, ovariectomized; ABH/OG, alcian blue hematoxylin/orange G. DPF, days post-fracture

ABH/OG staining revealed that proteoglycan-rich cartilage tissue emerged in both callus of sham mice and OVX mice from 5 DPF. At 14 DPF, cartilage tissue fully bridged the fracture gap, indicating the formation of cartilaginous callus. Then cartilaginous callus were gradually moved away and woven bone-like matrix formed at the edge of the callus. At 21 DPF, cartilage tissue of callus was almost completely resorbed in sham mice. However, cartilage tissue of callus in OVX mice was less at 14 DPF and was still obvious at 21 DPF. At 28 DPF, callus had remodeling of the callus was almost completed in sham mice while there was still plenty of spongy bone tissue in the callus of OVX mice (Fig. 2a). Histological quantification also confirmed increased cartilage tissue in the callus of OVX mice at 21 DPF (Fig. 2b, *p* < 0.05), which suggested a delay in endochondral ossification process during fracture healing in OVX mice.

### TNF-α and IL-6 but not IL-1β expression were impaired in OVX mice during fracture healing

The protein and gene expressions of inflammatory cytokines, including TNF-α, IL-1β and IL-6, in the fracture haematoma or callus of both sham and OVX mice were detected by immunohistochemical analysis and real-time PCR respectively.

**Fig. 3:**
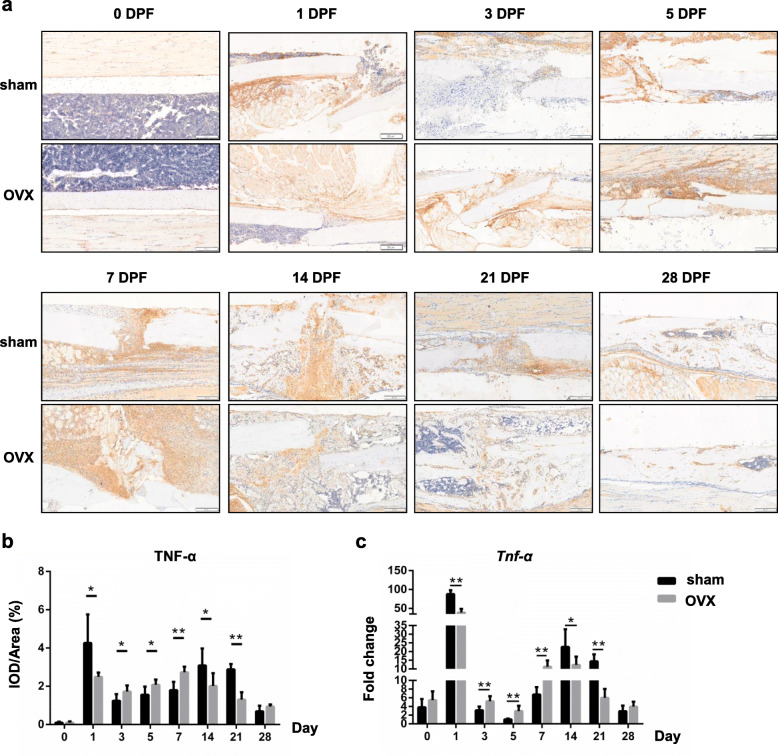
TNF-α expression in the OVX mice during fracture healing. **a**, **b** Immunohistochemistry staining and quantifications of TNF-α in the fracture haematoma/callus of sham and OVX mice at different time points of fracture healing. DPF, days post-fracture. Scale bars, 200 μm. **c** mRNA expressions of TNF-α in the fracture haematoma/callus at different time points were determined by real time PCR. *, *p* < 0.05; **, *p* < 0.01 compared with the sham group. *n* = 6/group

Before fracture, TNF-α protein was rarely found in the tibias of sham and OVX mice. In sham mice, expression of TNF-α was increased in the periosteum and bone marrow surrounding the fracture site at 1 DPF, and then was decreased at 3 DPF and 5 DPF. From 7 DPF, dramatic TNF-α expression was found in the callus and became even intensive and concentrated at 14 DPF, especially in the bridged gap. TNF-α expression was then gradually decreased from 21 DPF. In OVX mice, small amount of positive staining was found in the periosteum at 1 DPF. After a temporary reduction at 3 DPF, TNF-α expression was gradually increased again until 7 DPF. At 14 DPF when TNF-α expression in sham mice reached the peak, TNF-α expression in OVX mice began to decrease (Fig. 3a). The quantification result showed that, compared with that of sham mice, TNF-α expression in the callus of OVX mice were significantly lower at 1, 14, 21 DPF, and were higher at 3, 5, 7 DPF (Fig. 3b; 1, 3, 5 and 14 DPF, *p* < 0.05; 7 and 21 DPF, *p* < 0.01).

**Fig. 4:**
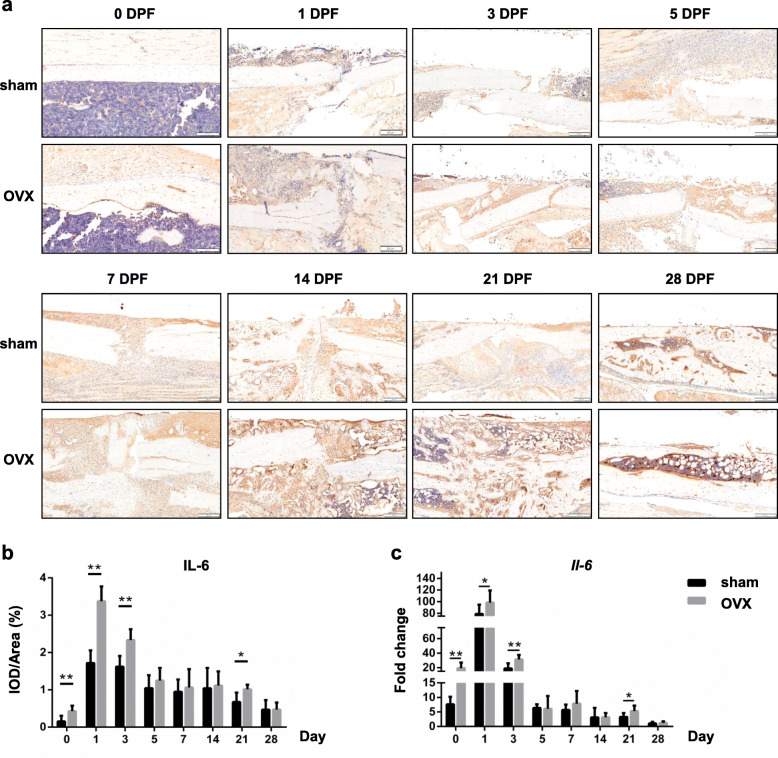
IL-6 expression in the OVX mice during fracture healing. **a**, **b** Immunohistochemistry staining and quantifications of IL-6 in the fracture haematoma/callus of sham and OVX mice at different time points of fracture healing. DPF, days post-fracture. Scale bars, 200 μm. **c** mRNA expressions of IL-6 in the fracture haematoma/callus at different time points were determined by real time PCR. *, *p* < 0.05; **, *p* < 0.01 compared with the sham group. *n* = 6/group

IL-6 protein was also rarely found in the tibias of sham mice before fracture. After fracture, plenty of IL-6 positive staining emerged in the surrounding tissue of fracture site, and became less gradually as the following fracture healing process went on. In OVX mice, IL-6 expression was always highly expressed, especially at the early stage of fracture healing (Fig. 4a). Quantification data also showed that the IL-6 expression level reached the peak as early as 1 DPF, and was gradually decreased to in a very slow rate. IL-6 expression in callus of OVX mice was significantly higher at 0, 1, 3 and 21 DPF (Fig. 4b; 0, 1 and 3 DPF, *p* < 0.01; 21 DPF, *p* < 0.05).

**Fig. 5:**
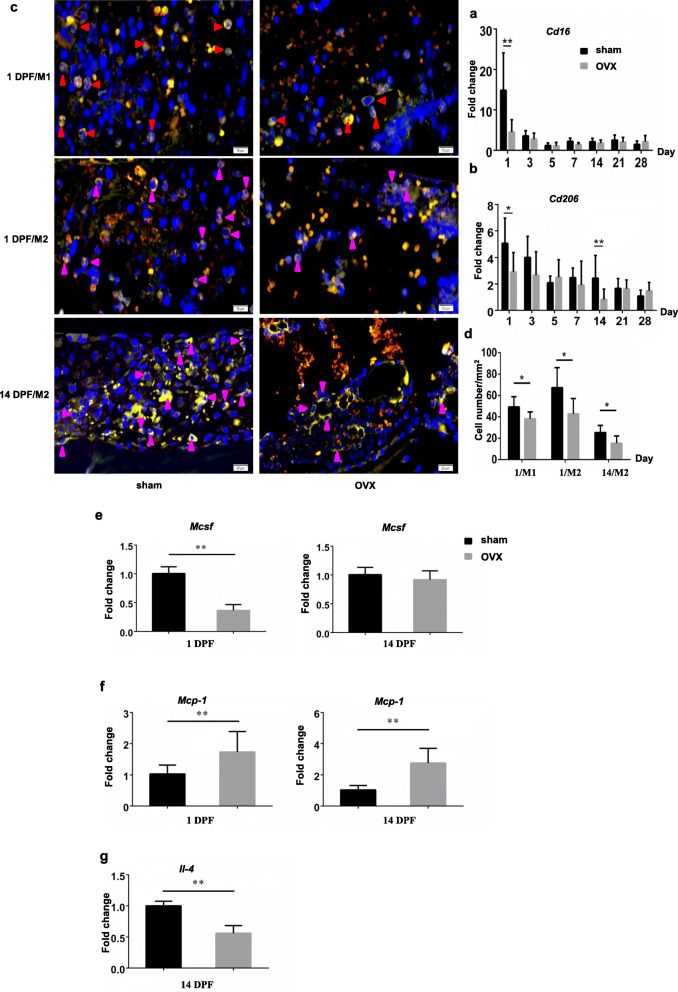
Impaired inflammatory macrophage formation in the OVX mice during fracture healing. **a**, **b** mRNA expressions of M1 (*Cd16*) and M2 (*Cd206*) macrophage specific markers in the fracture haematoma/callus of sham and OVX groups. *, *p* < 0.05; **, *p* < 0.01 compared with the sham group. *n* = 6/group. **c** Dual immunofluorescence staining for M1/M2 macrophages in the fracture haematoma/callus of sham and OVX mice. Blue, DAPI positive nucleus. Red arrow heads, M1 macrophages. Pink arrow heads, M2 macrophages. **d** Numbers of M1 and M2 macrophages in the fracture haematoma/callus of were quantified. *, *p* < 0.05 compared with the sham group. *n* = 6/group. **e**-**g** mRNA expressions of MCSF, MCP-1 and IL-4 in the fracture haematoma/callus of sham and OVX groups. **, *p* < 0.01 compared with the sham group. *n* = 3/group

We also examined the mRNA levels of these inflammatory genes in callus of sham and OVX mice. It was showed that there were two peaks (1 DPF and 14 DPF) in *Tnf-α* expression and one peak (1 DPF) in *Il-6* expression (Figs. 3c and 4c). *Tnf-α* expression was significantly decreased at 1, 14, 21 DPF, and increased at 3, 5 and 7 DPF (Fig. 3c; 1, 3, 5, 7 and 21 DPF, *p* < 0.01; 14 DPF, *p* < 0.05) in OVX mice. In addition, *Il-6* expression was increased significantly at 0, 1, 3 and 21 DPF (Fig. 4c;0 and 3 DPF, *p* < 0.01; 1 and 21 DPF, *p* < 0.05) in OVX mice.

However, no significant differences in both gene and protein expressions of IL-1β in callus was shown between sham and OVX mice, though IL-1β was highly expressed in callus after fracture (Additional Figure 2).

### Both inflammatory macrophages and osteal macrophages were impaired in OVX mice during fracture healing

Since the expressions of inflammatory cytokines during fracture healing were impaired in OVX mice, we assessed the changes in inflammatory macrophage status during fracture healing, referring to M1 macrophage and M2 macrophage which were the main sources of inflammatory cytokines. The expression levels of *Cd16* and *Cd206*, markers of M1 and M2 macrophage respectively, in the fracture haematoma or callus were also detected. *Cd16* expression was significantly decreased at 1 DPF only (Fig. 5a; 1 DPF, *p* < 0.01), and *Cd206* expression was decreased at 1 and 14 DPF in OVX mice (Fig. 5b; 1 DPF, *p* < 0.05; 14DPF. *p* < 0.01).

Dual immunofluorescence staining for F4/80 and iNOS was performed to detect M1 macrophages at fracture site as well as F4/80 and CD206 for M2 macrophages, and the results were consistent with the results of the expression levels of *Cd16* and *Cd206* (Fig. 5c and d). Compared with the sham mice, OVX mice showed less M1 and M2 macrophages in the fracture haematoma at 1 DPF, and also less M2 macrophages in the callus at 14 DPF (Fig. 5a), which was further confirmed by quantification results (Fig. 5d, *p* < 0.05). Gene expressions of some molecules involved in macrophage differentiation were also observed. The gene expression level of macrophage-colony stimulating factor (M-CSF) in the fracture haematoma of OVX mice was dramatically decreased compared with sham group at 1 DPF (Fig. 5e, *p* < 0.01), but no significant difference between these two groups at 14 DPF was revealed. Expression level of monocyte chemoattractant protein-1 (MCP-1) mRNA in the fracture haematoma or callus of OVX mice was significantly increased compared with sham group at both 1 DPF and 14 DPF (Fig. 5f, *p* < 0.01), while *Il-4* expression at 14DPF was decreased (Fig. 5g, *p* < 0.01).

**Fig. 6:**
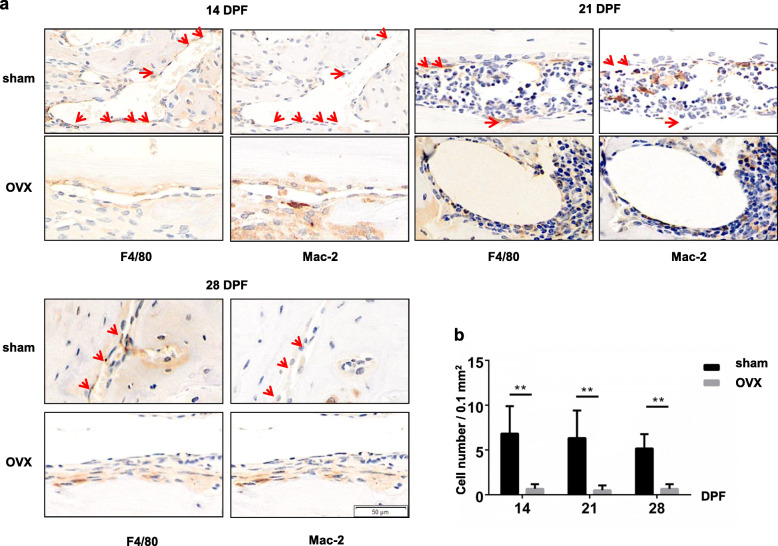
Impaired ostealmacrophage formation in the OVX mice during fracture healing. **a** Immunohistochemistry staining for F4/80^+^Mac-2^−^ macrophages with serial sections of the fracture haematoma/callus from OVX and sham mice. Arrows, macrophages with F4/80 positive staining and Mac-2 negative staining. **b** Quantifications of the numbers of F4/80^+^Mac-2^−^ macrophages within the periosteal callus or endochondral ossification callus. An average callus area of 0.1 mm^2^ was assessed. **, *p* < 0.01 compared with the sham group. *n* = 6/group

Osteal macrophages have been detected in multiple stages of fracture healing and been proved to contribute to fracture healing though regulating osteoblast functions [6]. Here we identified osteal macrophages from inflammatory macrophages through immunohistochemical staining for F4/80 and Mac-2 with series sections. Cells with positive F4/80 staining and negative Mac-2 staining were considered osteal macrophages (F4/80^+^ Mac-2^−^) [23]. In both sham and OVX mice, there were very few F4/80^+^Mac-2^−^ macrophages in the early stage of fracture healing (data not shown). More F4/80^+^Mac-2^−^ macrophages emerged on the surface of newly formed bone marrow cavities until 14 and 21 DPF when endochondral ossification occurred within the callus. As callus remodeling went on, F4/80^+^Mac-2^−^ macrophages became to decrease again (Fig. 6a). These findings were consistent with the results of previous study [7]. Being different from that of sham mice, F4/80^+^Mac-2^−^ macrophages could rarely be detected in the middle and late stage of fracture healing in OVX mice (Fig. 6a). The differences in F4/80^+^Mac-2^−^ macrophage numbers between sham mice and OVX mice were confirmed by quantification (Fig. 6b, *p* < 0.01).

## Discussion

The expressions of inflammatory cytokines during fracture healing follow a biphasic pattern, with a transient peak in the initial inflammatory phase, and a second peak during chondrocyte maturation and endochondral ossification [1, 24]. Being different from those secreted by inflammatory cells in the initial phase, the inflammatory cytokines expressed during chondrocyte maturation and endochondral ossification are mainly synthesized by osteoblasts, chondrocytes and other cells, which can result the second peak in the middle and later stage of fracture [25, 26]. In murine fracture models, inflammatory response of TNF-α, IL-1β and IL-6 peak 24 h following the fracture injury and decline to baseline levels rapidly by 72 h. Thereafter, a second peak in TNF-α, IL-1β expression occur approximately 14 days following the fracture injury [24, 27].

Fracture healing comprises two bone repair processes: intramembranous and endochondral bone formation. The roles of inflammatory cytokines in intramembranous bone formation can be summarized into two aspects. On one hand, inflammatory cytokines acted as chemokines which were responsible for bone marrow stromal cell (bMSC) recruitment [28]. On the other hand, inflammatory cytokines could promote intramembranous bone formation directly by inducing bMSCs osteogenic differentiation [29]. Endochondral bone formation includes a series of processes such as cartilage proliferation, maturation, hypertrophy and apoptosis, and inflammatory cytokines also play an important role [30]. Clinical studies confirmed that inflammatory cytokines such as TNF-α, IL-1β and IL-6 were highly expressed in osteoarthritis, which induced chondrocyte apoptosis and destroy articular cartilage [31, 32]. TNF-α could promote apoptosis of chondrocytes by up-regulating the apoptotic genes in chondrocytes [33], and chondrocyte apoptosis and resorption of the mineralized cartilage during the endonchondral period were delayed in TNF-α receptor deficient mice [34].

In our study, TNF-α expression was dysregulated during fracture healing in OVX mice. Reduced transient expression in the initial inflammation phase and endochondral ossification phase might lead to decreased abilities in bMSC recruitment and osteogenic differentiation as well as chondrocyte maturation and endochondral ossification. Furthermore, TNF-α expression in OVX mice was consistently up-regulated at other time points except for the initial inflammation phase and endochondral ossification phase of fracture healing. TNF-α has dual roles on osteogenic differentiation depended on the exposure time. Huang et al. [29] reported that long-term treatment of TNF-α induced inhibitory effect on osteogenic differentiation in vitro while short-term promoted osteogenic differentiation. Also TNF-α was proved with strong ability to induce osteoclast formation [35]. Therefore, consistent up-regulated TNF-α expression in OVX mice could contribute partially to decreased trabecular bone number and sparser structure of callus, which was consistent with previous studies [17, 36].

IL-6 levels in the plasma and fracture callus peaked as early as 6 h after fracture, and progressively declined to a low level in 72 h. Compared with sham mice, plasma IL-6 expression in OVX mice was even higher at 6 h after fracture [37], which might due to the increase of neutrophils [38]. In this study, IL-6 expression in OVX mice was increased before fracture, and the increase was even obvious during the early stage of fracture which was consistent with the previous finding. Furthermore, there was almost no significant difference in IL-6 expression between OVX and sham mice during the middle and late stages of fracture healing. IL-6 is a potential inducer of osteoclast differentiation independent of receptor activator of nuclear factor kappa-B ligand (RANKL) [39], and can be produced by plenty of cells under physiological and pathological conditions including immune-mediated cells, mesenchymal cells, vascular endothelial cells, fibroblasts, and so on. Unlike TNF-α, the action of IL-6 signaling is dependent on the availability of RANKL in the microenvironment [40].

However, no change in IL-1β expression between sham mice and OVX mice was found. This was also consistent with previous animal study that during the fracture healing of IL-1β receptor (I*lr1*^−/−^) mice, no differences in callus, cartilage or bone matrix production could be found [41].

Previous studies have demonstrated macrophages presented in multiple stages of fracture healing [7, 8]. Inflammatory macrophages were observed in the early anabolic events of femoral fracture healing; the phenotype changed during the first 3 days from predominantly M1 to M2, and M2 macrophages dominated the gap area at 7 days. Osteal macrophages were predominantly activated at day 14 to 21 after fracture [7, 42]. In our study, both M1 and M2 macrophages were predominantly presented in the initial phase, and osteal macrophages presented in the middle and late stage during fracture healing in sham mice. OVX led to deceased M1 and M2 macrophages in the initial phase and rarely detected osteal macrophages in the middle and late stages of fracture healing. Moreover, OVX mice also showed fewer M2 macrophages in the endochondral ossification phase of fracture healing. M-SCF has been confirmed a significant contributor to differentiation and maintenance of macrophage multiple populations [43], and is typically used in vitro to differentiate human monocytes into macrophages [44]. Therefore, the dramatically decreased expression of M-SCF at 1 DPF in OVX mice may partially contributed to the loss of both M1 and M2 macrophages in the initial phase of bone fracture. IL-4 is capable of stimulating M2 polarization, while MCP-1 is an inhibitory factor for M2 polarization from bone marrow-derived macrophages [45, 46]. Increased expression of MCP-1 and decreased expression of IL-4 could also partially explain the phenotype of M2 macrophage reduction at the early and middle phases of fracture healing.

M1 macrophages can secrete pro-inflammatory cytokines such as TNF-α, promoting angiogenesis and recruiting bMSCs. M2 macrophages participate in phagocytosing apoptotic cells, dissolving thrombus and promoting tissue repair [47]. In addition, M2 macrophages were reported a higher angiogenic potential compared to other macrophage subsets [48]. In vitro experiment also evidenced increased osteogenic markers expression and bone mineralization of M2 macrophage co-cultured bMSCs [49]. Therefore, decreased inflammatory macrophages as well as lower expressions of inflammatory cytokines, especially TNF-α, disturbed the initiation inflammatory responses of fracture healing, and deceased M2 macrophages could also impaired angiogenesis, osteogenesis, and endochondral ossification in OVX mice. Osteal macrophages were predominantly presented within the maturing/remodeling hard callus during fracture; depletion of osteal macrophages during bone healing has been demonstrated impaired endochondral callus formation and osteoblast mineralization using the Mafia transgenic mouse model [7, 8, 50]. However, the exact molecular mechanisms by which osteal macrophages affect callus remodeling have not been fully addressed.

There were still some limitations of this study. Firstly, we only examined the expressions of several critical cytokines due to limited samples obtained from mice models, including TNF-α, IL-1β and IL-6. There were plenty of inflammatory or pro-inflammatory cytokines presenting in the process of fracture healing, and more studies should be carried out to totally reveal the expression patterns of these cytokines. Secondly, activated resident macrophages including osteal macrophages, are able to shift into pro-inflammatory M1 macrophages or anti-inflammatory M2 macrophages by different cytokines [51]. Therefore, the cell shift between inflammatory macrophages and osteal macrophages continuously occurred during the process fracture healing. Ovariectomy leads to a chronic increase in systemic inflammation, and it may probably result in dysregulation of the cell shift. However, this issue was not confirmed in the current study. Thirdly, due to the limited sample size, mechanical test which could contribute the evaluation of the quality of fracture healing was not included in this study. Lastly, we selected a small sample size at each time point since it was the first time to evaluate the changes in macrophages and inflammatory cytokine expressions during fracture healing between sham and OVX mice, which might limit greater extrapolation of our results.

## Conclusions

Taken together, ovariectomy in mice led to irregular macrophages and inflammatory cytokine expressions during fracture healing, especially in the initial inflammatory phase and the endochondral ossification phase, which might impair fracture healing in OVX mice. It was suggested that irregular inflammatory responses might occur during fracture healing process of postmenopausal women.

## Supplementary Information

**Additional file 1: Figure 1.** Radiographic changes of the tibias in sham and OVX mice at different time points of fracture healing.

**Additional file 2: Figure 2.** Immunohistochemistry staining and real-time PCR for the protein and mRNA expressions of IL-1β in the fracture haematoma/callus of sham and OVX mice.

## Data Availability

the datasets used and/or analyzed during the current study are available from the corresponding author on reasonable request.

## Abbreviations

TNF-α: Tumor necrosis factor-α
IL-1β: Interleukin-1β
IL-6: Interleukin-6
OVX: Ovariectomized
DPF: Days post-fracture
E2: Estradiol
PCR: Polymerase chain reaction analysis
TV: Total volume
BV: Bone volume
ABH/OG: Alcian blue hematoxylin/orange G
iNOS: Inducible nitric oxide synthase
HRP: Horseradish peroxidase
DAPI: 4′,6-diamidino-2-phenylindole
M-CSF: Macrophage-colony stimulating factor
IL-4: Interleukin-4
MCP-1: Monocyte chemoattractant protein-1
bMSC: Bone marrow stromal cell
RANKL: Receptor activator of nuclear factor kappa-B ligand
