# Supplementary figures and images for: Changes in macrophage and inflammatory cytokine expressions during fracture healing in an ovariectomized mice model
Source: BMC Musculoskelet Disord. 2021 May 28;22:494. doi: 10.1186/s12891-021-04360-z (PMC8164289; doi:10.1186/s12891-021-04360-z)

## Slide 1
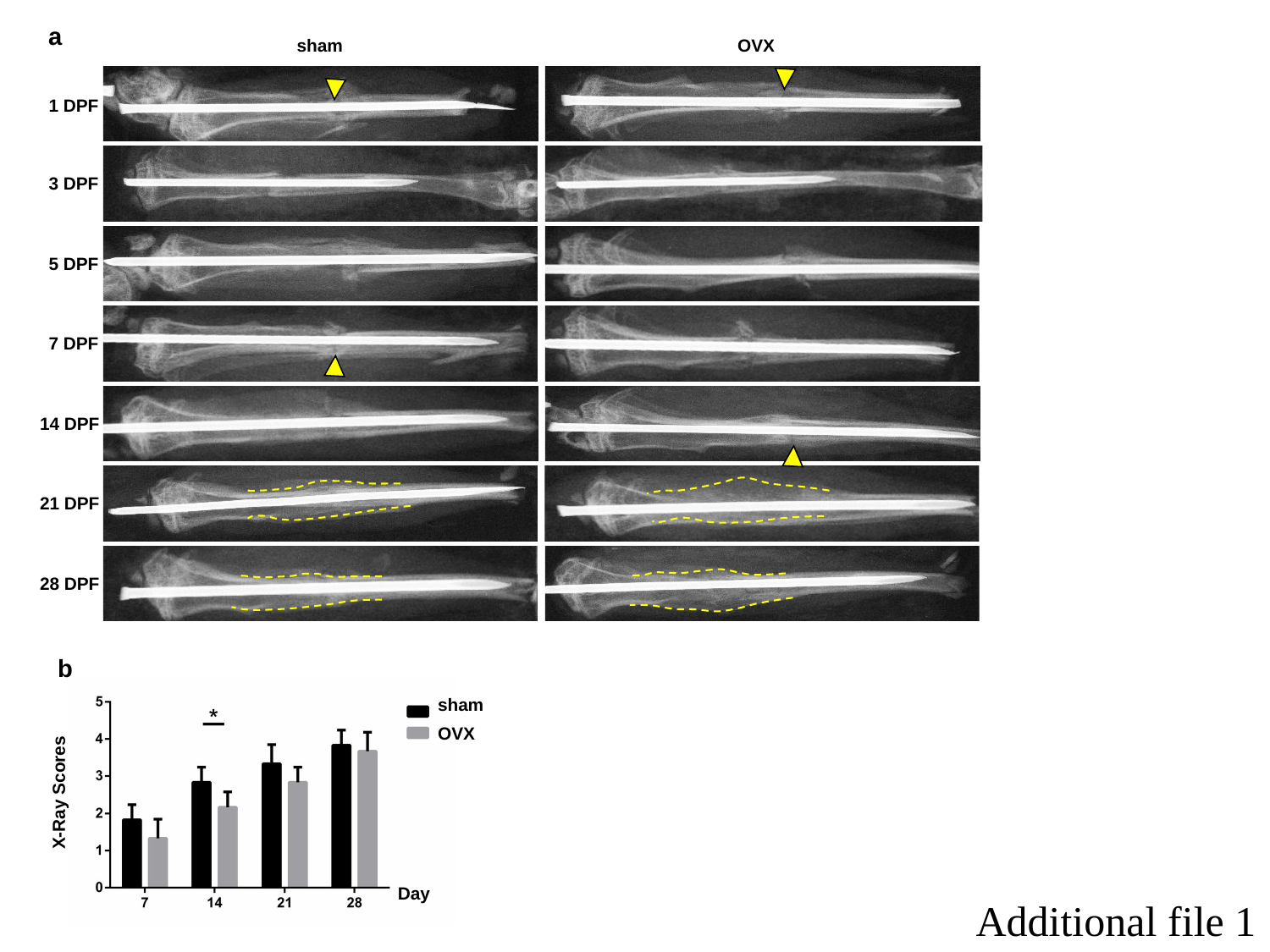

a
sham
OVX
1 DPF
3 DPF
5 DPF
7 DPF
14 DPF
21 DPF
28 DPF
X-Ray Scores
b
sham
OVX
Day
Additional file 1

Supplement: Supplementary file 1 — Additional file 1: Figure 1. Radiographic changes of the tibias in sham and OVX mice at different time points of fracture healing. [file 12891_2021_4360_MOESM1_ESM.ppt]
