# Supplementary material for: Changes in macrophage and inflammatory cytokine expressions during fracture healing in an ovariectomized mice model
Source: BMC Musculoskelet Disord. 2021 May 28;22:494. doi: 10.1186/s12891-021-04360-z (PMC8164289; doi:10.1186/s12891-021-04360-z)
Supplement: Supplementary file 2 — Additional file 2: Figure 2. Immunohistochemistry staining and real-time PCR for the protein and mRNA expressions of IL-1β in the fracture haematoma/callus of sham and OVX mice. [file 12891_2021_4360_MOESM2_ESM.ppt]

## Slide 1
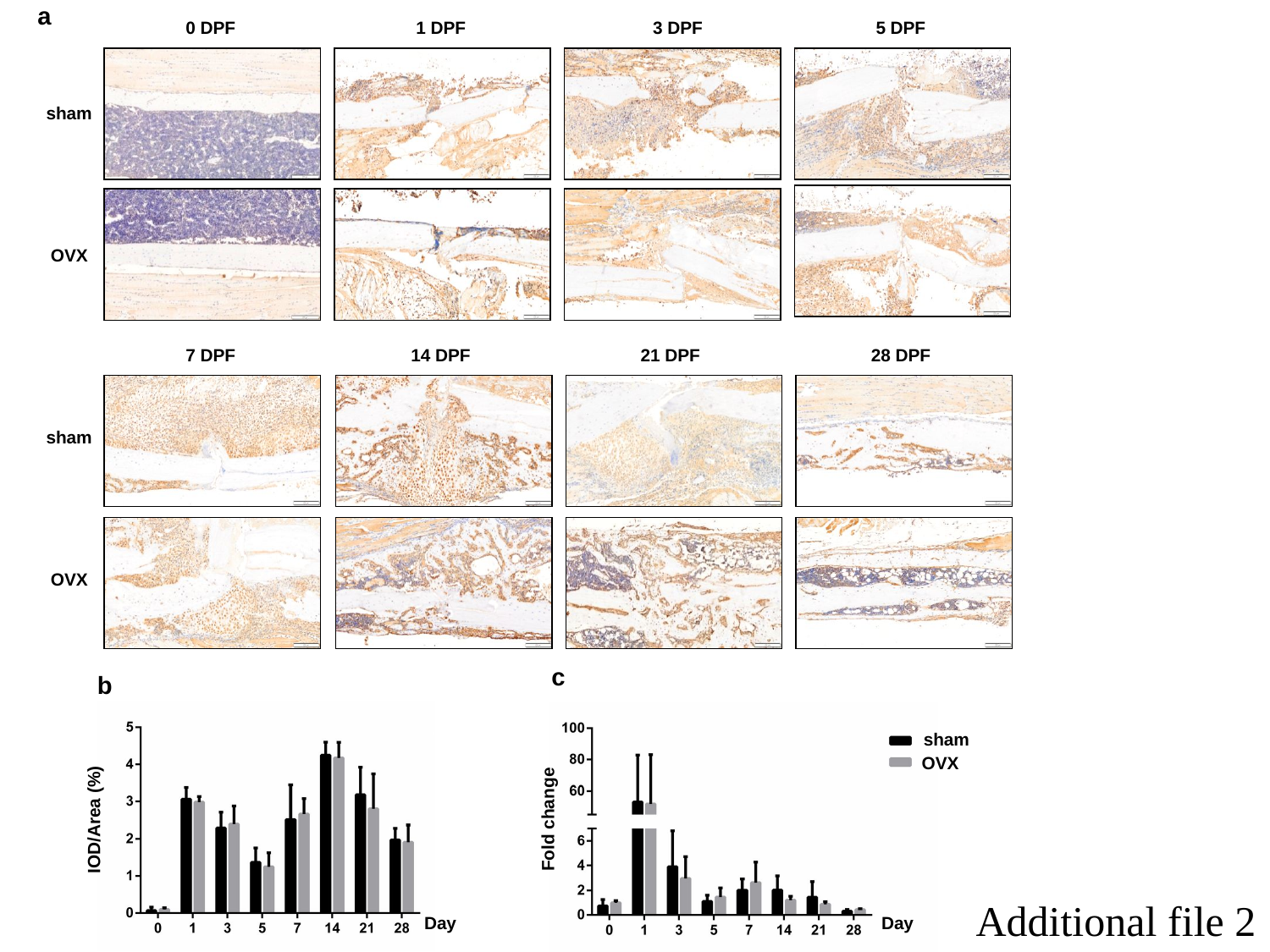

0 DPF
1 DPF
3 DPF
5 DPF
a
sham
OVX
7 DPF
14 DPF
21 DPF
28 DPF
sham
OVX
IOD/Area (%)
Fold change
c
b
sham
OVX
Additional file 2
Day
Day
